# Prediction of six macrophage phenotypes and their IL-10 content based on single-cell morphology using artificial intelligence

**DOI:** 10.3389/fimmu.2023.1336393

**Published:** 2024-01-04

**Authors:** Mischa Selig, Logan Poehlman, Nils C. Lang, Marita Völker, Bernd Rolauffs, Melanie L. Hart

**Affiliations:** ^1^G.E.R.N. Research Center for Tissue Replacement, Regeneration & Neogenesis, Department of Orthopedics and Trauma Surgery, Faculty of Medicine, Medical Center—Albert-Ludwigs-University of Freiburg, Freiburg im Breisgau, Germany; ^2^Department of Biosystems Science and Engineering, ETH Zürich, Basel, Switzerland

**Keywords:** artificial intelligence, single-cell morphology, cell shape, macrophage phenotype, IL-10, inflammation, macrophage, fingerprint

## Abstract

**Introduction:**

The last decade has led to rapid developments and increased usage of computational tools at the single-cell level. However, our knowledge remains limited in how extracellular cues alter quantitative macrophage morphology and how such morphological changes can be used to predict macrophage phenotype as well as cytokine content at the single-cell level.

**Methods:**

Using an artificial intelligence (AI) based approach, this study determined whether (i) accurate macrophage classification and (ii) prediction of intracellular IL-10 at the single-cell level was possible, using only morphological features as predictors for AI. Using a quantitative panel of shape descriptors, our study assessed image-based original and synthetic single-cell data in two different datasets in which CD14+ monocyte-derived macrophages generated from human peripheral blood monocytes were initially primed with GM-CSF or M-CSF followed by polarization with specific stimuli in the presence/absence of continuous GM-CSF or M-CSF. Specifically, M0, M1 (GM-CSF-M1, TNFα/IFNγ-M1, GM-CSF/TNFα/IFNγ-M1) and M2 (M-CSF-M2, IL-4-M2a, M-CSF/IL-4-M2a, IL-10-M2c, M-CSF/IL-10-M2c) macrophages were examined.

**Results:**

Phenotypes were confirmed by ELISA and immunostaining of CD markers. Variations of polarization techniques significantly changed multiple macrophage morphological features, demonstrating that macrophage morphology is a highly sensitive, dynamic marker of phenotype. Using original and synthetic single-cell data, cell morphology alone yielded an accuracy of 93% for the classification of 6 different human macrophage phenotypes (with continuous GM-CSF or M-CSF). A similarly high phenotype classification accuracy of 95% was reached with data generated with different stimuli (discontinuous GM-CSF or M-CSF) and measured at a different time point. These comparably high accuracies clearly validated the here chosen AI-based approach. Quantitative morphology also allowed prediction of intracellular IL-10 with 95% accuracy using only original data.

**Discussion:**

Thus, image-based machine learning using morphology-based features not only (i) classified M0, M1 and M2 macrophages but also (ii) classified M2a and M2c subtypes and (iii) predicted intracellular IL-10 at the single-cell level among six phenotypes. This simple approach can be used as a general strategy not only for macrophage phenotyping but also for prediction of IL-10 content of any IL-10 producing cell, which can help improve our understanding of cytokine biology at the single-cell level.

## 1 Introduction

Macrophages are heterogeneous populations of cells and, in response to microenvironmental cues, exhibit a broad spectrum of polarized phenotypes. Simplified, two extremes of polarized macrophages include the classically activated pro-inflammatory M1 macrophages and the alternatively activated anti-inflammatory M2 macrophages. However, it is now appreciated that macrophage polarization is more complex and this oversimplified approach does not adequately describe the broad phenotype spectrum of macrophages. Depending on the microenvironmental stimuli and activation state, macrophages can be further divided into subsets such as M0, M1, M2a, M2b, M2c, and M2d that reflect functional differences ranging from homeostatic, anti-/pro-inflammatory to anti-fibrotic/fibrotic and tissue repair phenotypes (1–5).

Macrophage phenotypic characterization is typically assessed by standard techniques such as flow cytometry, ELISA, RT-PCR, and western blot. Another method of investigating macrophage properties is via quantification of cell morphology (6–11). Several studies have shown a correlation between cell shape and macrophage activation (7–11). Once activated, cells in general, including macrophages, adapt not only phenotypically but also morphologically to their microenvironment due to changes in cytoskeletal dynamics, which in turn can affect the shape and the function of a given cell (6, 8, 12, 13). Thus, morphological profiling offers a high-throughput, low cost, and high-dimensional method of biological readouts that can potentially be used to understand phenotypic responses of macrophages to microenvironmental cues.

As of recent, a few studies have used artificial intelligence (AI)-based approaches to classify the macrophage activation state and phenotype at the single-cell level (9, 11, 14–16). Nonetheless, the majority of AI-based models were assembled using the RAW264.7 murine immortalized macrophage cell line (11, 15, 16), which considerably differs from human cells in morphology, gene and protein regulation and expression, immunometabolism and immunological responses to TLR4 signaling (17–21). Because AI models are data-driven, it is imperative in human medicine to perform predictive investigations on human cells to not only assess the accuracy of predictions in human cell-based experiments but more importantly, for possible application in clinically relevant situations. Whereas image-based AI using morphological features for differentiating between M1 vs. M2 macrophages has been investigated in one study using human peripheral blood monocytes (9), it was not applied to M2 macrophage subsets. While often broadly referenced as having an anti-inflammatory functions, there are prominent functional distinctions between M2a and M2c subtypes (3, 22). Using morphological assessments to accurately classify not only M1 vs. M2 but also M2a vs. M2c activations states could be useful in many settings.

IL-10 is a pleiotropic cytokine that has a fundamental role in modulating inflammation and maintaining cell and tissue homeostasis (23). Flow cytometry is typically used to measure the intracellular expression of IL-10 and studies have indeed used flow cytometry to investigate the intracellular expression of IL-10 in monocytes and polarized macrophages as well as other cell types (24–28). However, automated high-throughput image analysis of single-cell morphology has not been used for intracellular cytokine detection or for prediction of intracellular IL-10 at the single-cell level via AI, e.g. by using morphology as a predictor.

By combining cell imaging with a computational image analysis pipeline, here we tailor an automated high-throughput approach (13, 29–33) for single-cell morphological profiling of various human macrophage populations. Specifically, we focus on imaging cell morphology and intracellular IL-10 to assess the responsiveness and effector potential of these cells under different polarizing conditions. Using a novel high throughput approach that combines the use of both image-based original and synthetic single-cell data, we determined that cell shape can distinguish M0, M1, M2a, and M2c macrophage subtypes and accurately classify a cell’s immunogenic profile by classifying intracellular IL-10 content. Our findings demonstrate a new image-based macrophage feature classification method on the single-cell level for the accurate classification of phenotype and IL-10 production, which could be widely used to predict both macrophage phenotypes and, more generally, the functional response of any IL-10-producing cell type in response to microenvironmental cues.

## 2 Methods

### 2.1 Isolation of monocytes using CD14

Peripheral blood mononuclear cells (PBMCs) were collected from three to five (indicated in each figure legend) healthy human blood donors by venipuncture in EDTA-coated vacutainer tubes (Sarstedt). Due to the fact that blood was only obtained from the authors, according to our local ethics committee (University of Freiburg Ethics Committee), under the relevant national and local regulations, ethical approval and informed consent was not needed. PBMCs were separated from other blood components by Ficoll-Plaque (GE Healthcare Life Sciences) density gradient centrifugation and resuspended in MACs buffer containing anti-CD14 microbeads (Miltenyi Biotec). The isolation was performed via positive selection using the MS MACs Column (Miltenyi Biotec) and the MiniMACs magnet (Miltenyi Biotec) according to the manufacturer’s protocol. The CD14+ monocytes were counted and seeded at a density of 50,000 cells/ml in RPMI-1640 cell medium (Sigma Aldrich) containing 10% FBS (Bio Chrome) and PenStrep (Life Technologies Corporation). The CD14+ monocytes were treated with maturation factors GM-CSF (10 ng/mL, Peprotech) or M-CSF (25 ng/mL, Peprotech) to induce M1 or M2 macrophages, while M0 macrophages were left untreated. The cell suspensions were placed in T25 flasks (Greiner Bio-One) for two days.

### 2.2 CD14 staining for FACS purity and vitality assessment

After MACs isolation, a portion of the monocyte suspension was used for FACS assessment. Monocytes were pelleted and resuspended in the cold (4°C) FACS buffer, PBS (Sigma Aldrich) containing 0.5% BSA (Sigma Aldrich), and 0.1% Sodium Azide (Sigma Aldrich). To assess the purity of CD14-positive cells, 100 μl of the cell suspension was stained with 5 μl APC-Cy7 mouse anti-human CD14 (BD Pharmingen). For cell vitality, 1 μl of Ghost Dye Blue 516 (Tonbo) was added to the cell suspension. The cell suspension was then incubated in the dark at 4°C for 30 minutes. After incubation, the cell suspension was centrifuged at 400 g for 5 minutes and washed with 500 μl of FACs buffer. This was repeated three times before being resuspended in 200μL of FACS Buffer, transferred to FACs tubes, and kept on ice. FACS samples were analyzed using the BD LSR Fortessa (BD Biosciences) flow cytometer. APC-Cy7 was excited at 650 nm and emission measured at 785 nm, Blue 516 was excited at 488 nm with emission measured at 516 nm. Compensation was unnecessary because the chosen staining APC-Cy7 and Ghost dye blue had minimal spectra overlap. Data was processed using FlowJo 9.9.6 (FlowJo, LLC, Ashland, OR).

### 2.3 Monocyte activation for macrophage phenotype differentiation

**Figure 1:**
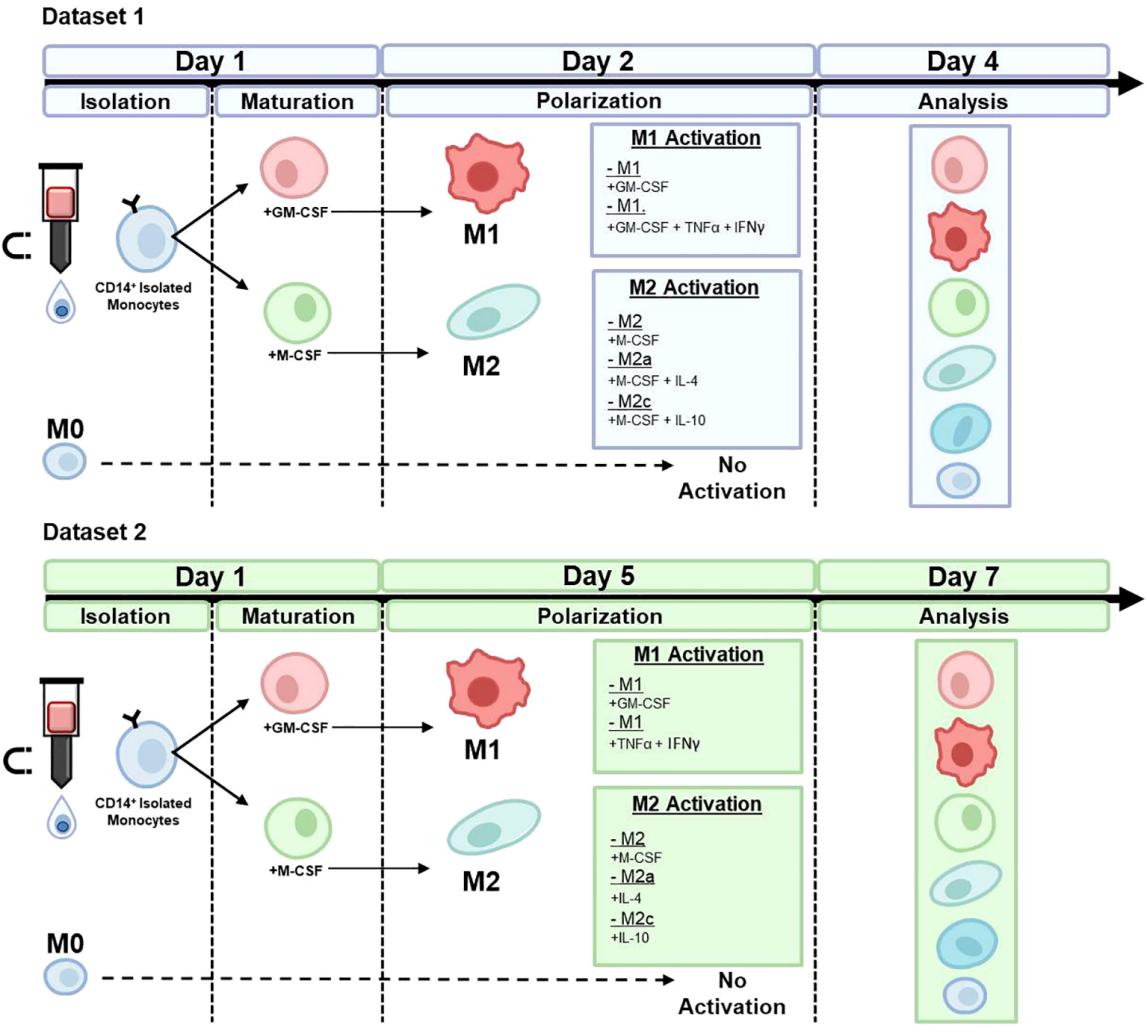
Macrophage phenotype differentiation protocols for dataset 1 and dataset 2. CD14+ monocytes were isolated using MACs and maintained in media to serve as a non-treatment M0 control group, whereas the other cells were polarized in the presence of specific polarizing agents in the presence of either continuous GM-CSF or M-CSF (Dataset 1) or, with the exception of M0, absence of GM-CSF or M-CSF (Dataset 2; validation of RF machine learning model).

Established cytokines were used to generate distinct phenotypic macrophage states to mimic different *in vivo* situations (3, 5, 34–37). CD14+ monocytes were maintained in media to serve as a non-treatment M0 control group, whereas the other cells were first matured in either GM-CSF or M-CSF and then polarized with specific polarizing agents in the presence of continuous GM-CSF or M-CSF (Dataset 1) or absence of continuous GM-CSF or M-CSF (Dataset 2). **Figure 1** gives an overview of the differences in stimuli and time points between Datasets 1 and 2. Thus, for Dataset 1, the following groups were assessed at day 4: M0, GM-CSF-M1, GM-CSF/TNFα/IFNγ-M1, M-CSF-M2, M-CSF/IL-4-M2a, and M-CSF/IL-10-M2c. For Dataset 2, which was used for validation of the here used AI approach, the following groups were assessed at day 7: M0, GM-CSF-M1, TNFα/IFNγ-M1, M-CSF-M2, IL-4-M2a, and IL-10-M2c.

In detail, the CD14+ monocytes were seeded at 1x10^6^ cells/mL cell density in a 96-well plate (Greiner Bio One). M0 cells were maintained in media containing RPMI-1640 cell medium (Sigma Aldrich) containing 10% FBS (Bio Chrome) and PenStrep (Life Technologies Corporation), which served as a control in both datasets. After maturation with either GM-CSF (10 ng/ml, Peprotech) or M-CSF (25 ng/ml, Peprotech), cells were polarized into M1 or M2 phenotypes using the same concentrations of GM-CSF or M-CSF (referred to GM-CSF-M1 or M-CSF-M2, in both datasets). Media was then supplemented with standard cytokines to prompt activation into divergent macrophage phenotypes. Activated M1 macrophages were generated through supplementing RPMI media with 10 ng/mL TNF-α (R&D) and 10 ng/mL IFN-γ (Peprotech) in the presence of continuous GM-CSF (GM-CSF/TNFα/IFNγ-M1, Dataset 1) or discontinuous GM-CSF (TNFα/IFNγ-M1, Dataset 2) to obtain pro-inflammatory M1 macrophage populations. Distinct M2 subtypes were generated through the addition of IL-4 (10 ng/mL, Peprotech) for M2a macrophages or IL-10 (10 ng/ml, Peprotech) for M2c cells in the presence of continuous M-CSF (M-CSF/IL-4-M2a; M-CSF/IL-10-M2c; Dataset 1) or discontinuous M-CSF (IL-4-M2a; IL-10-M2c; Dataset 2).

### 2.4 ELISA for IL-6, IL-10 and TNF-α protein quantification

ELISA for targets human IL-6, IL-10, and TNF-α (R&D Systems Europe Ltd) was performed according to the manufacturer’s protocol using 100 µl medium supernatant. Optical density was measured at 450 nm with a NANOstar Spectrometer (Thermo Fisher Scientific).

### 2.5 Cell staining and microscopy

Cells were first fixed using 4% paraformaldehyde (PFA). This was followed by a wash and permeabilization step using 1% Triton X-100 (Carl Roth) for 30 min at room temperature. Possible unspecific antibody binding sites were blocked using 2% BSA (Sigma Aldrich). CD163 and CD80 staining was used to validate the macrophage polarized phenotypes. For CD163, CD80, and IL-10 staining, the cells were incubated with specific primary antibodies (rabbit anti-human CD163 mAb, Abcam ab182422; mouse anti-human CD80 mAb, Invitrogen, 16080985; and rabbit anti-human IL-10 mAb, Abcam ab215975) overnight at 4°C. The following day, the wells were washed and a staining solution, which included secondary antibodies (goat anti-mouse IgG (H+L) cross-adsorbed secondary antibody AlexaFluor 568 (A-11004, 1:1000, Thermo Fisher) for CD80; goat-anti rabbit IgG (H+L) cross-adsorbed secondary antibody AlexaFluor 488 (A-11008, 1:500, Thermo Fisher) for CD163, phalloidin (to visualize F-actin; A-30105, 1:400), and DAPI (0.1 µg/ml, D8417-5mg, Sigma-Aldrich) was applied in DPBS for two hours. Then, fresh DPBS was supplied, and microscopical images, captured from random fields of view within each well, with a 10x magnification were taken with the Axio Observer Z1 microscope (Zeiss Oberkochen, Germany).

### 2.6 High-throughput quantitative measurements of single-cell macrophage morphology, CD163, CD80 and IL-10 protein expression

Single macrophage analysis was performed using a Fiji-based (38) single-cell shape analysis algorithm that we previously used to phenotype differentiated mesenchymal stromal cells (MSCs) (6, 30–33) and healthy vs. inflamed and degenerating chondrocytes (13, 29–33). The fluorescent staining with DAPI and phalloidin visualized the cell’s nucleus and body (F-actin). After staining, the image analysis algorithm segmented and separated individual cells from the image background by assigning pixels in the image to either the cell or the image background based on their intensity values and calculated watershed distance maps based on the distance between cell nuclei. The segmentation created binary image maps, with the cells represented in white and the image background in black. Upon successful segmentation and cell separation, the algorithm proceeded to identify and detect single cells within these binary image maps and, from that, calculate individual shape descriptor values.

Single-cell morphology was assessed by calculating the following panel of shape descriptors: area of the single cells (μm^2^), length (major axis [μm]), width (minor axis [μm]), circularity (4*π(area/perimeter^2^), aspect ratio (ratio of major to the minor axis, used an indicator of cell elongation), roundness (4*area/(π*major axis length^2^) and solidity (are/convex area(cell)). To clarify, length is different from aspect ratio, which is the ratio between the length and width of a cell. It increases if the length continuously increases while the width decreases or remains stagnant. The descriptors circularity and roundness are relatively insensitive to irregular boundaries, unlike solidity, which is quantified as the ratio of the cell area to the area of a convex hull of the cell. A solidity value of 1 indicates a solid cell, and less than 1 indicates a cell with an irregular boundary or containing holes. Single-cell protein expression was measured as the cellular raw integrated intensity of background-subtracted images, which is the pixel sum of the values of the detected fluorescent intensity. To allow for different exposure times during image acquisition, the intensity values for single-cell CD163, CD80, and IL-10 were normalized to fluorescent bead intensity standard curves (linear calibration curves) that were calculated from the emission of fluorescent beads at specific exposure times.

### 2.7 Synthetic dataset generation with the ‘SuperTiles’ algorithm

We recently introduced the ‘SuperTiles’ algorithm to generate synthetic data from image tile data (39). Here, we used the algorithm to generate a synthetic data set on morphology and protein-based cell features with the goal of improving the classification accuracy of macrophage phenotypes through increased data set size. The algorithm was implemented in Python 3.9 and iteratively selected random data subsets (entire data rows) from the same macrophage class. Each of the parameters of the selected subsets was averaged (aggregated) into a single synthetic data point and the newly calculated synthetic data points together built a new synthetic data row. In more simple terms, the algorithm randomly selected single cells and their features and averaged these selected single cells into one aggregated SuperTile. This means the number of generated synthetic cells increased with the randomly selected number of cells and their sample time per iteration of the SuperTiles algorithm to enhance the synthetic dataset. The algorithm used two key parameters: the amount of sampled data rows (t) of each individual cell and its attributed features (i.e, all metrics (image-based features) for all cells), whereas the sample time (s) described how often a given number of random data rows was sampled. In this study, ‘t’ was set from 2 to 40 (for morphology and protein features) and from 2 to 100 (for morphology features alone). A value for ‘s’ >1 indicated dataset bootstrapping with data replacement. During bootstrapping, selected data points were aggregated. Here, ‘s’ was set from 5 to 40. Balanced synthetic datasets were generated using the formula n_SuperTiles_=n_class_size (minority class)_ ∗ s/t. Therefore, the size of the synthetic dataset generated was dependent on the original dataset (i.e., original total cell number). For example, in the present study, there were less M0 cells vs. M1 control cells. Hence, the algorithm created more synthetic M0 cell data to balance the final numbers of all classes in the final dataset used for training the random forest algorithm. The newly calculated synthetic dataset was then split into training and test sets (70/30) for predictive modeling, as described below.

### 2.8 Random forest classification of macrophage phenotype

We used a random forest (RF) algorithm (40) as described in our prior study (41) to classify macrophage phenotype (class) using cell morphology alone vs. cell morphology and protein intensity levels as predictor variables. RF model training and testing were implemented in Python 3.9 via the “pycaret” (42) package. The data was normalized for algorithm training, and all parameters were considered equally weighted model features. Each RF model was trained with 10-fold cross-validation, for which the dataset was split into training and test subsets (70/30).

We used the following RF modelling performance indicators: (i) accuracy, which indicates the number of correct predictions/total number of predictions; (ii) AUC (Area Under the Curve), which measures the area underneath the ROC (Receiver Operating Characteristics) curve of TPR (true positive rate) against FPR (false positive rate (sensitivity)) with an AUC=1 indicating the correct classification of all samples; (iii) recall, which equals TPR; (iv) precision=TP (True Positive)/(TP + FP (False Positive)); (v) F1 score, which is the harmonic mean of precision and recall (TPR) with F1=(precision ∗ recall)/(precision + recall); (vi) the kappa score for quantifying model prediction with a lower score indicating better model performance (score = (probability of agreement – the probability of random agreement)/(1 – the probability of random agreement); (vii) the Matthews Correlation Coefficient (MCC), which quantifies the quality of binary or multiclass classification by calculating the correlation between true and predicted values, and which we used in a prior study (41).

### 2.9 Statistical analysis

The data was analyzed using SigmaPlot v.14.0 (Systat, Chicago) and Microsoft Excel (v. 2020). First, the normality of the data was tested (Kolmogorov-Smirnov-test). For comparing two groups, normally distributed data was subjected to the Student’s t-test and non-normal distributed data was analyzed using the Mann-Whitney-Rank-Sum-test. An ANOVA on Ranks test was performed to compare more than two statistical groups with non-normal distributed data. If the ANOVA revealed significant differences between the groups, a *post-hoc* test (Dunn’s Method) was used for multiple comparisons between two groups because the Dunn’s test allowed comparing groups with unequal sample size. Correlation analyses were performed using the “R” (43) packages “Hmisc” (44) and “corrplot” (45). The Spearman Rank Order correlation method was used if one or more variables were categorical. The Pearson product moment correlation test was used when variables were numerical. For correlation analyses, the M0 class was coded as 0, the M1 control as 1, the M1 Stim as 2, the M2 control as 3, the M2a class as 4, and the M2c type as 5. To visualize data covariance between macrophage morphology and protein expression, a clustered image map (CIM) was generated. This map visualizes scaled and centered data with a color code whose key indicates the standard deviations away from the mean of each feature, whereas dendrograms indicate Euclidian distance-based hierarchical clustering. We performed multivariate projection-based modeling (PLS) on the dataset, specifically PLS-DA, which is an adaptation developed to classify categorical data. The CIM and PLS-DA analyses were performed with the “mixOmics” (46) package in “R”. Statistical differences were considered significant for p<0.05.

## 3 Results

**Figure 2:**
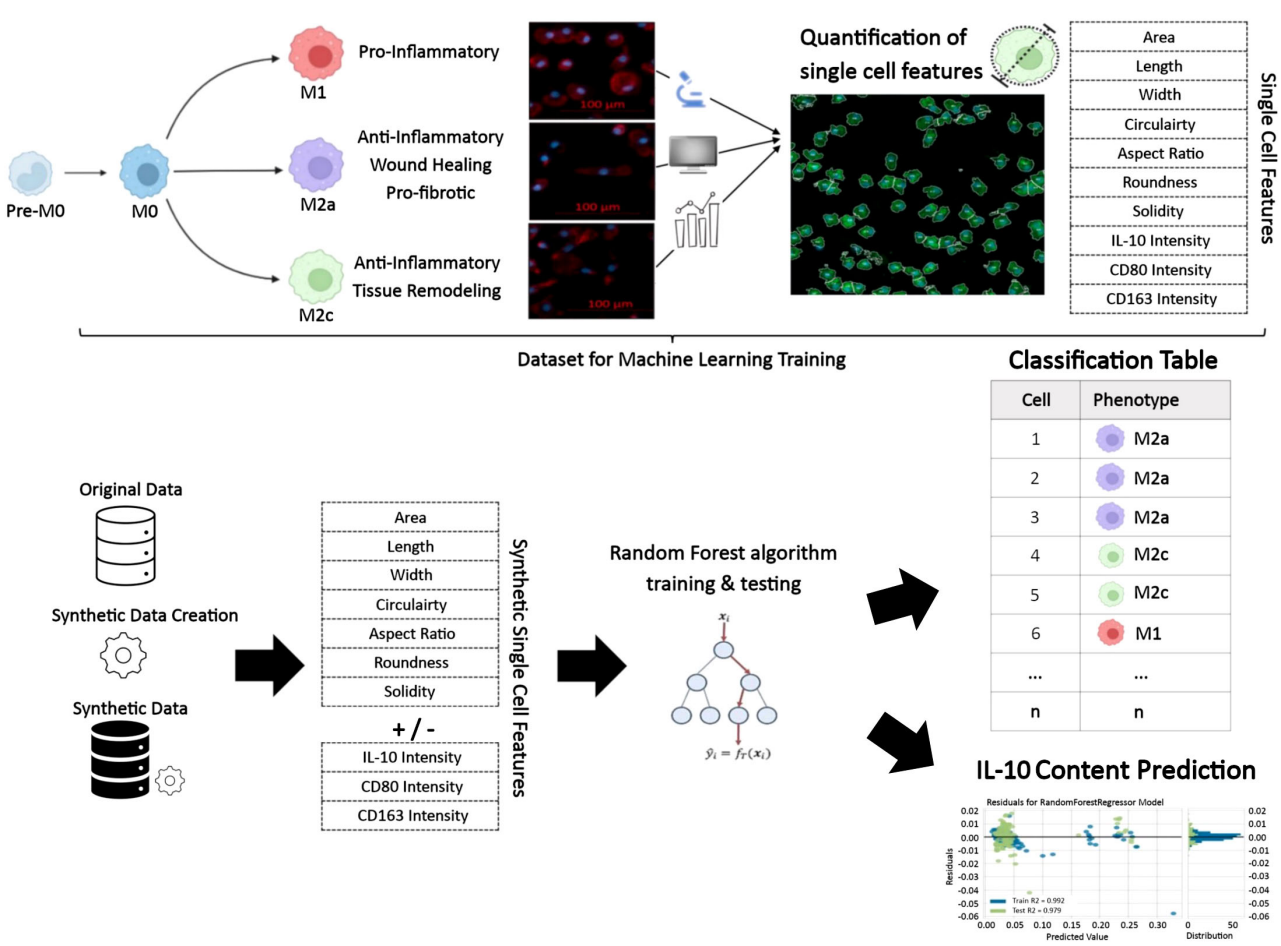
Illustration of the workflow for prediction of macrophage phenotypes and intracellular IL-10 based on single-cell morphology alone or in combination with protein intensities using artificial intelligence. This approach is applicable for profiling monocyte/macrophage phenotypes under other conditions and, in the case of IL-10, may be applied to other IL-10 producing cell types.

An overview of the methods, which allowed classification of the macrophage phenotypes and their IL-10-producing potential, based on single-cell morphology using machine learning, is provided in **Figure 2**.

### 3.1 Isolation of pure CD14+ positive cells from human PBMCs

**Figure 3:**
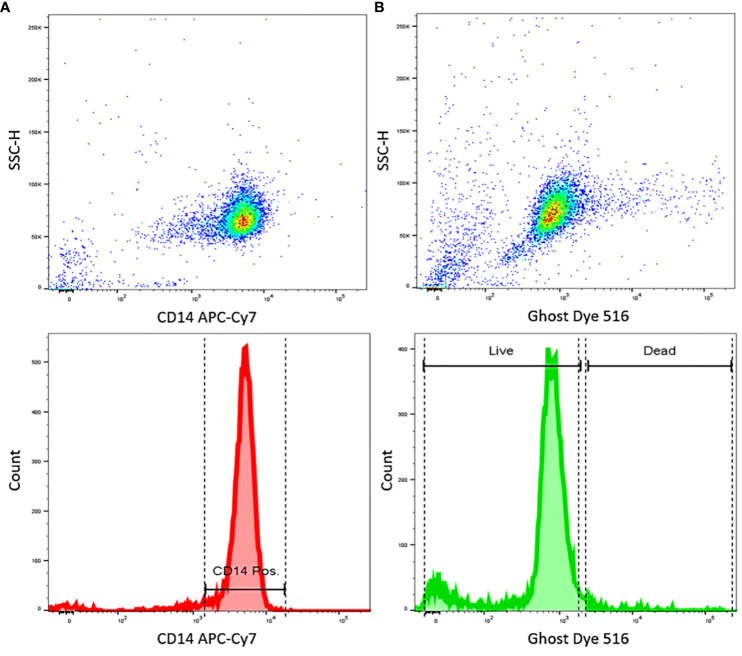
FACS results show highly pure and vital CD14+ cells isolated from human PBMCs. **(A)** Dot plot shows the dispersion of measurements of CD14 staining intensity, and the histogram showing of CD14 staining intensity, indicating a high purity of CD14 positive cells. **(B)** Dot plot shows the dispersion of measurements of Ghost dye staining intensity, and the histogram shows Ghost dye intensity, indicating highly viable cells. Data representative of n=3 different donors.

As a first step, human CD14+ blood-derived monocytes isolated from PBMCs were assessed by flow cytometry for purity and cell vitality. Staining with ghost dye confirmed a vital cell population. Monocyte population purity was over 95% (**Figure 3**), consistent with data in the literature using similar MACs techniques (9, 47).

### 3.2 Protein expression profiles following polarization of monocyte-derived macrophages

**Figure 4:**
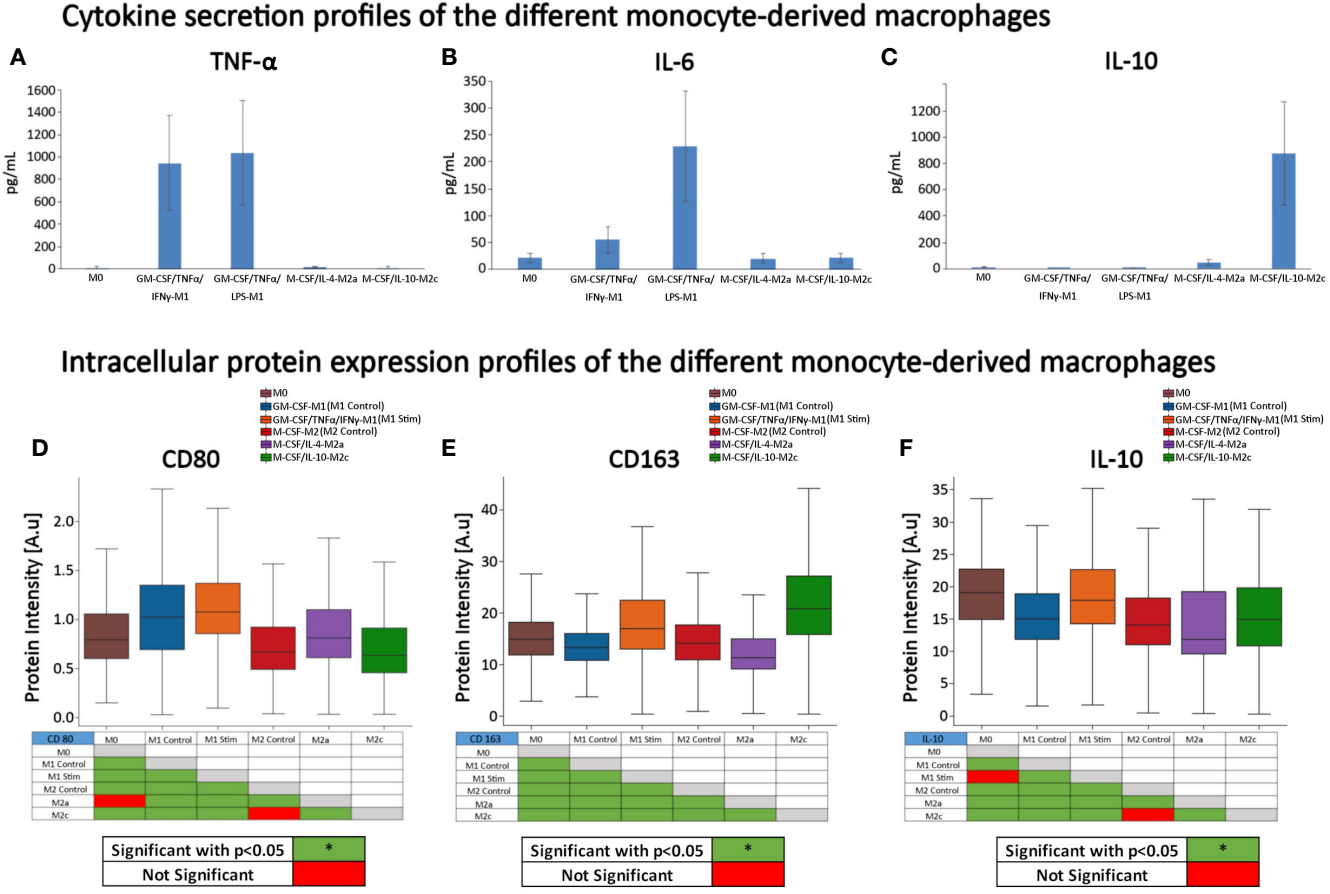
Cytokine secretion and protein expression profiles of the different monocyte-derived macrophages. **(A)** TNF‐*α*, **(B)** IL-6, and **(C)** IL-10 secretion, and **(D)** CD163, **(E)** CD80, and **(F)** IL-10 intensity in the presence of continuous GM-CSF or M-CSF. **(A–C)** n=5 per group from 5 different donors on day 4 after maturation and polarization. Data is representative of the mean protein secretion +/- SEM. **(D–F)** expression profiles (protein intensity) of surface receptor proteins CD80, CD163, and intracellular IL-10 were quantified using fluorescent microscopy image-based analysis; data is based on n = 353 (M0), 3078 (GM-CSF-M1 control), 1891 (GM-CSF/TNFα/IFNγ-M1), 1321 (M-CSF-M2 Control), 1077 (M-CSF/IL-4-M2a), and 1584 (M-CSF/IL-10-M2c) individual cells analyzed of n = 3 experiments per group, using 3 different donors. Boxplots: the boxes define the 25th and 75th percentiles, the central line indicates the median, and error bars define the 10th and 90th percentiles. *p<0.05. Red-green tables indicate significant differences calculated with ANOVA on Ranks tests and *post-hoc* pairwise comparisons (Dunn’s Method).

First, we performed ELISA to quantify the extracellular protein production of IL-10, IL-6, and TNF-α to validate the phenotypic profile of the cells after polarization. After 4 days of maturation and polarization, the culture supernatant was used for quantification of IL-6, TNF-α and IL-10 (**Figures 4A–C**) and the cells were fluorescently stained to analyze their CD163, CD80, and IL-10 intracellular protein expression (**Figures 4D–F**). The profiles of the different types of macrophages confirmed that cells were polarized into the corresponding macrophage states. As expected, GM-CSF-M1 and GM-CSF/TNFα/IFNγ-M1 polarized cells resulted in a M1‐like pro‐inflammatory phenotype with increased secretion of TNF-α and IL‐6 and increased CD80 expression. Stimulation with IL‐10 resulted in an M2c‐like phenotype with increased anti-inflammatory CD163 expression and the highest IL-10 secretion compared to all other groups. This data is consistent with the secretion (47–49) and flow cytometry CD marker expression profiles (47, 50–53) from other studies using similar polarization protocols.

Since we aimed to determine if cell morphology could predict macrophage phenotypes and intracellular IL-10, we additionally quantified intracellular IL-10 intensities. M0 and GM-CSF/TNFα/IFNγ-M1 cells expressed the highest intracellular IL-10 protein intensities, followed by M-CSF/IL-10-M2c macrophages. Conversely, M-CSF/IL-4-M2a macrophages exhibited the lowest IL-10 intensity (**Figure 4F**). When comparing secreted IL-10 (**Figure 4C**) vs. intracellular IL-10 expression (**Figure 4F**), differences were noted, suggesting that the cells with the highest intracellular IL-10 protein expression were not the cells that secreted the most IL-10.

### 3.3 Morphological differences between polarized macrophages

**Figure 5:**
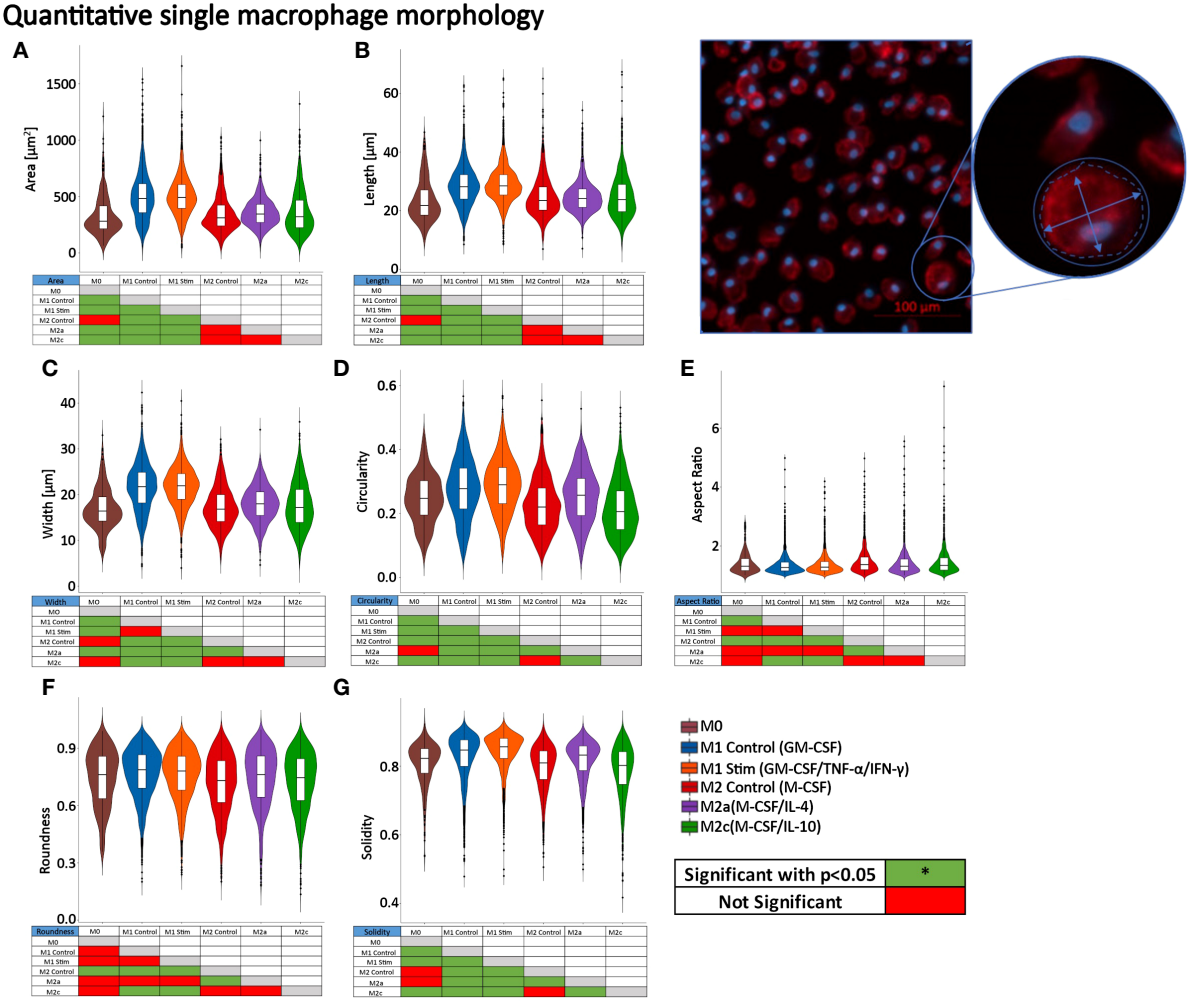
Single-cell macrophage morphology differs significantly between macrophage phenotypes. Cell morphometric measurements of **(A)** area, **(B)** length, **(C)** width, **(D)** circularity, **(E)** aspect ratio, **(F)** roundness, and **(G)** solidity of n=3 different donors with n = 353 (M0), 3078 (GM-CSF-M1), 1891 (GM-CSF/TNFα/IFNγ-M1), 1321 (M-CSF-M2), 1077 (M-CSF/IL-4-M2a), and 1583 (M-CSF/IL-10-M2c) individual cells measured. Violin plots visualize data distribution. Outliers are visualized as black dots above the 95th or below the 5th percentiles. *p<0.05. Boxplots within the violin plots: the boxes define the 25th and 75th percentiles, the central line indicates the median, and error bars define the 10th and 90th percentiles. *p<0.05. Red-green tables indicate significant differences calculated with ANOVA on Ranks tests and *post-hoc* pairwise comparisons (Dunn’s Method).

To determine if there were quantitative significant differences in shape descriptors (area, length, width, circularity, aspect ratio, roundness, and solidity), single-cell macrophage analysis was performed using a Fiji-based analysis algorithm (13, 29). When comparing different groups of macrophages, the violin box plots (**Figure 5**) revealed that the GM-CSF/TNFα/IFNγ-M1 (largest) and GM-CSF-M1 macrophages were larger in cell area than the other groups. The M0 control group had the smallest cell area, followed by M-CSF-M2, M-CSF/IL-4-M2a, and M-CSF/IL-10-M2c. The M1 (both GM-CSF/TNFα/IFNγ-M1 and GM-CSF-M1) macrophages were similar in shape, except for their cell width and aspect ratio. The M2 macrophages had a similar area and length, with M-CSF/IL-4-M2a’s being wider, more circular, elongated, rounder, and solid than M-CSF-M2 and M-CSF/IL-10-M2c cells. The M-CSF/IL-10-M2c cells were similar in shape to the M-CSF-M2 macrophages. These cell morphometric results are in line with previous studies showing that M1 macrophages are larger and more round and M2 macrophages are more elongated (7, 10). Overall, these results demonstrated that macrophage phenotypes differed in morphology, suggesting that a quantitative analysis of single macrophage morphology via high-throughput and automated image analysis algorithms may be a useful method for identifying shape differences between the different phenotype classes of macrophages.

### 3.4 A clustered image map showing individual response patterns of macrophage subtypes in cell morphology descriptors and protein intensities

**Figure 6:**
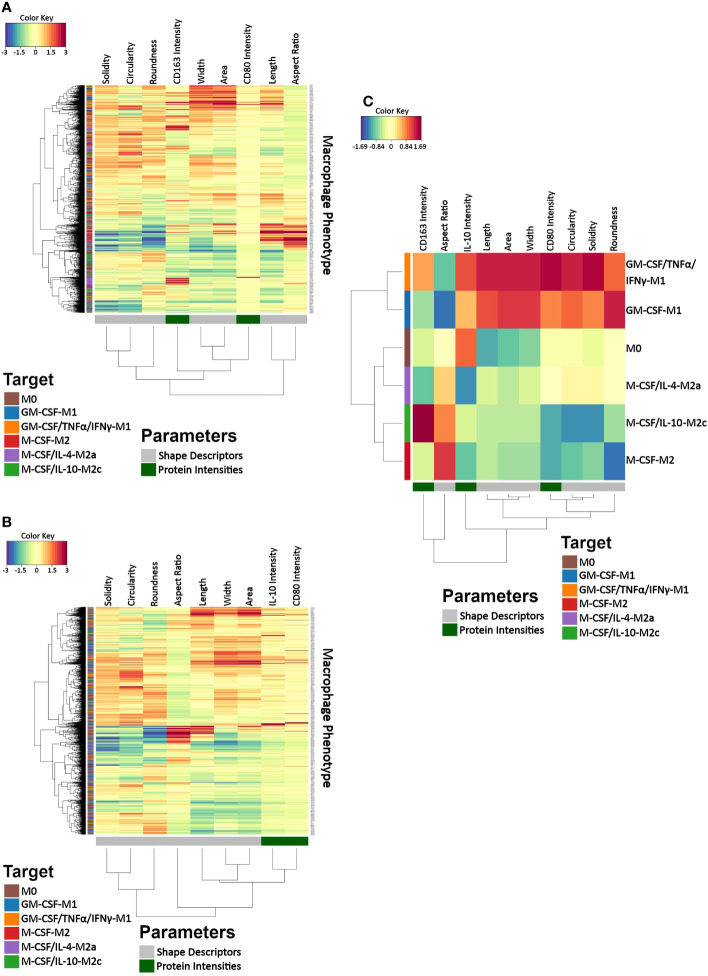
Clustered image maps (CIMs) for visualizing data co-variation of morphology and protein intensities as a function of macrophage class. **(A, B)** Two CIMs were calculated on the single-cell level, which differed in the depicted protein features because our setup allowed determining 4 microscope channels in parallel. The top CIM depicts cell morphological features calculated with phalloidin and DAPI channels as well as channels for CD80 and CD163, whereas the lower CIM depicts IL-10 and CD80 in addition to cell morphological features (phalloidin, DAPI). The two CIMs on the individual cell level revealed the complexity of the data set and demonstrated that the cell features depicted no easily recognizable response pattern relative to the induced macrophage classes. This was in contrast to the average value CIM **(C)**, which demonstrated distinct macrophage feature clustering according to the induced phenotype: M0, GM-CSF-M1, GM-CSF/TNFα/IFNγ-M1, M-CSF-M2, M-CSF/IL-4-M2a, and M-CSF/IL-10-M2c. A CIM visualizes scaled and centered data with a color code indicates the standard deviations away from the mean of each feature, whereas the dendrograms indicate clustering. The level of the parameters of a given category and their intensity of the red color denotes the number of standard deviations above the overall mean across all samples, and the intensity of the blue color denotes the number of standard deviations below the overall mean.

To explore co-variation among macrophage morphology and protein intensities related to the induced macrophage classes, we created CIMs on the single-cell level showing the individual cell’s response patterns in cell morphology descriptors and CD163 intensity (**Figure 6A**) and in cell morphology and CD80 and IL-10 intensities (**Figure 6B**) and another CIM with feature averages calculated for each macrophage class (**Figure 6C**). The single-cell CIM indicated the size of the generated data set was very large and was not helpful for identifying specific patterns or clusters relative to the induced macrophage classes. This was important because it revealed the complexity of the data set (**Figures 6A, B**), which we, in turn, used as motivation for the subsequent use of AI for classification. The horizontal dendrogram of the CIM depicting average values for each macrophage class (**Figure 6C**) revealed a clear hierarchical clustering for the feature averages: the two induced M1 classes (GM-CSF-M1 and GM-CSF/TNFα/IFNγ-M1) clustered together, as did the M0 and the M2a (M-CSF/IL-4-M2a) classes and also the M-CSF-M2 and M-CSF/IL-10-M2c classes. Moreover, the two induced M1 classes were clustered into one class and the M0, M2, M2a, and M2c classes were clustered into a second class. These hierarchical clustering results indicate how the feature values of macrophage classes contributed to overall similarities and dissimilarities between classes. Thus, the average value CIM demonstrated feature response patterns for the macrophage classes and a clear hierarchical clustering for the feature averages but not on the single-cell level, which motivated us to use AI for subsequent classification.

### 3.5 RF classification of macrophage classes solely based on cell morphology vs. cell morphology combined with protein intensities

**Figure 7:**
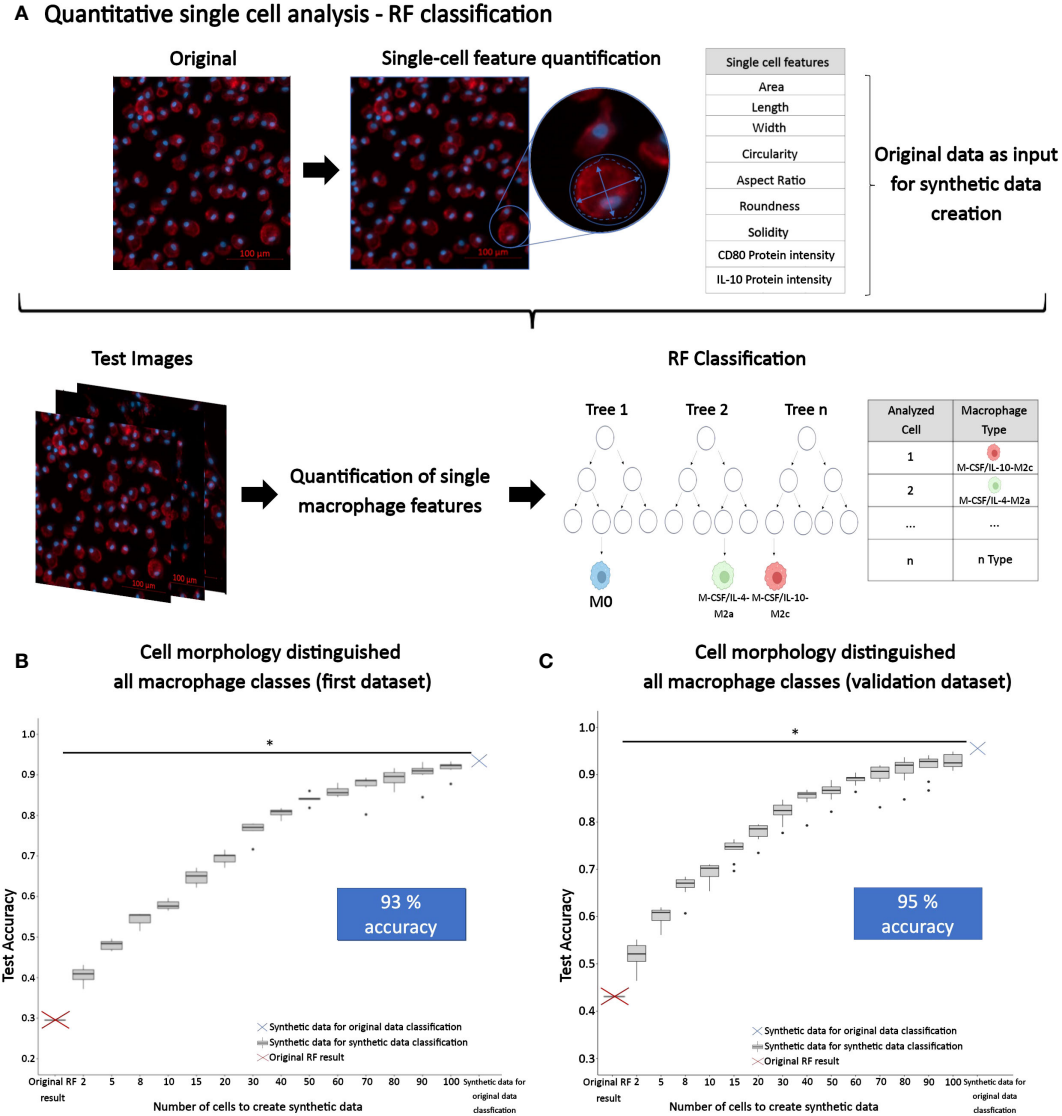
Quantitative single macrophage phenotyping. **(A)** Quantitative image analysis of macrophages was performed to quantify single macrophage morphology descriptors, as well as CD80 and IL-10 protein intensities. These features were used to train a RF prediction model for classifying the macrophage subtype. The original RF result was generated using 9304 cells in total consisting of n = 353 (M0), 3078 (GM-CSF-M1), 1891 (GM-CSF/TNFα/IFNγ-M1), 1321 (M-CSF-M2), 1077 (M-CSF/IL-4-M2a), and 1584 (M-CSF/IL-10-M2c) individual cells analyzed of n = 3 experiments per group, using 3 different donors. **(B)** Synthetic data was created to increase the original training set and test the resulting accuracy. For the first dataset (Dataset 1), this approach led to a final accuracy of 93%. This synthetic dataset consisted of 936221 SuperTiles in total with n = 155950 (M0), 155985 (GM-CSF-M1), 156020 (GM-CSF/TNFα/IFNγ-M1), 156055 (M-CSF-M2), 156090 (M-CSF/IL-4-M2a), and 156121 (M-CSF/IL-10-M2c) SuperTiles. **(C)** The approach to classify phenotype class with macrophage morphology features alone and in conjunction with synthetic data was validated with a second dataset (Dataset 2) that was generated using different stimuli and time points. This dataset yielded a 95% final accuracy, which indicated validation. The second synthetic dataset consisted of 1174248 SuperTiles in total with n = 195373 (M0), 195507 (GM-CSF-M1), 195641 (GM-CSF/TNFα/IFNγ-M1), 195775 (M-CSF-M2), 195909 (M-CSF/IL-4-M2a), and 196043 (M-CSF/IL-10-M2c) SuperTiles. *p<0.05, indicating a signifcant increase in classification accuracy with increased synthetic dataset size (i.e., 2 vs. 40 tiles).

To discriminate macrophage classes based on image-based cell features, we employed RF machine learning classification. Here, we utilized RF modeling with cell morphology features alone or combined with protein intensities as predictors to classify macrophage class (phenotypes) as shown in **Figure 7A**, for which the original data set was split into training and test sets. To test the resulting RF model accuracy as a function of data set size, we also used synthetic data that we generated from the original data (Dataset 1) with our ‘SuperTiles’ algorithm (39).

Using only macrophage morphological features of the original data (Dataset 1), RF classified the M1 (GM-CSF-M1) vs. M2 (M-CSF-M2) control classes with 92% accuracy (**Table 1**). Using only macrophage morphological features from the original data (Dataset 1) for classifying the M-CSF/IL-4-M2a vs. M-CSF/IL-10-M2c phenotype led to 63% accuracy; adding the CD80 and CD163 intensities increased the accuracy to 72% (**Table 1**). However, at this point of the study, when using only the original data (Dataset 1), we achieved for the classification of all six macrophage phenotypes low accuracies of 30% with only morphology features as predictors and 37% with morphology and protein intensity features as predictors (**Figures 7B, C**). A summary of all RF model classification accuracies and performance indicators is given in **Table 1**.

**Table 1:** Summary of RF classification model performance to classify macrophage phenotypes using original and synthetic datasets.

Original data (Dataset 1)								
RF Classification	Predictors	Accuracy	AUC	Recall	Precision	F1	Kappa	MCC
All macrophage phenotypes	**Morphology + CD80 + IL-10 intensities**	0.3725	0.7143	0.3725	0.4042	0.3816	0.2241	0.2258
GM-CSF-M1 vs. M-CSF-M2		0.9219	0.9676	0.8839	0.9514	0.9164	0.8433	0.8453
M-CSF-M2 vs. M-CSF/IL-4-M2a vs. M-CSF/IL-10-M2c		0.4611	0.6546	0.4611	0.4608	0.4599	0.1876	0.1888
GM-CSF-M1 vs. GM-CSF/TNFα/IFNγ-M1		0.6211	0.6619	0.5608	0.5016	0.5296	0.214	0.2149
M-CSF/IL-4-M2a vs. M-CSF/IL-10-M2c		0.7184	0.794	0.7269	0.7846	0.7546	0.4254	0.4271
All macrophage phenotypes	**Morphology alone**	0.2951	0.6468	0.2951	0.3306	0.3078	0.1294	0.1305
M-CSF-M2 vs. M2a vs. M-CSF/IL-10-M2c		0.4067	0.5887	0.4067	0.4152	0.4092	0.1077	0.1082
GM-CSF-M1 vs. GM-CSF/TNFα/IFNγ-M1		0.5667	0.5673	0.4815	0.4368	0.4581	0.0986	0.0989
M-CSF/IL-4-M2a vs. M-CSF/IL-10-M2c		0.6333	0.6861	0.6282	0.7205	0.6712	0.2611	0.2642
Synthetic data (from original Dataset 1)								
All macrophage phenotypes (validation dataset)	**Morphology alone**	**0.9313**	0.9451	0.9313	0.9276	0.9288	0.6772	0.6774
Synthetic data for validation (from original Dataset 2)								
All macrophage phenotypes (original dataset)	**Morphology alone**	**0.9585**	0.9541	0.9785	0.9565	0.9572	0.7103	0.7104

Predictors are indicated in bold. This data highlights that quantitative single-cell morphology alone can predict 6 different human macrophage phenotypes with a high accuracy in two different datasets (as shown in bold, Dataset 1: 93% accuracy; Dataset 2: 96% accuracy), generated with different stimuli and assessed at a different time point.

As a next step, we created synthetic data using our SuperTiles algorithm to increase the training dataset size. This allowed testing whether the increase in dataset size would increase classification accuracy. This step was important because an increase of dataset-size dependent accuracy would indicate in turn that the original dataset used for generating the synthetic dataset was phenotype class-specific but simply not large enough. Alternatively, if an increase in dataset size would not result in increased accuracy, this would indicate that the original dataset used for generating the synthetic dataset was not phenotype class-specific, and, in brief, not good enough. Interestingly, the increase in training data size resulted in a significant increase in RF classification accuracy: with only morphology features as predictors, we achieved with synthetic data a classification accuracy of 93% for classifying all 6 macrophage phenotypes (**Figure 7B**), which was a pronounced improvement of the 30% classification accuracy that was achieved by using the original data (Dataset 1) on morphology features. Thus, the original dataset (Dataset 1) used for generating the synthetic dataset was phenotype class-specific but simply not large enough, and increasing dataset size via generating synthetic data improved the classification accuracy of six phenotype classes by 63% to 93%. Importantly, this was achieved by training with synthetic data for classifying original data. The increase in classification accuracy of synthetic data with increased synthetic dataset size was significant when we compared the accuracies at 2 vs. 40 tiles in synthetic data (p<0.05). Overall, this is the first study to show that six macrophage phenotypes including M2 macrophages, particularly M-CSF-M2, M-CSF/IL-4-M2a, and M-CSF/IL-10-M2c subtypes, can accurately be distinguished from one another by their morphology.

### 3.6 Validation of the RF approach to classify macrophage classes based on cell morphology alone

**Figure 8:**
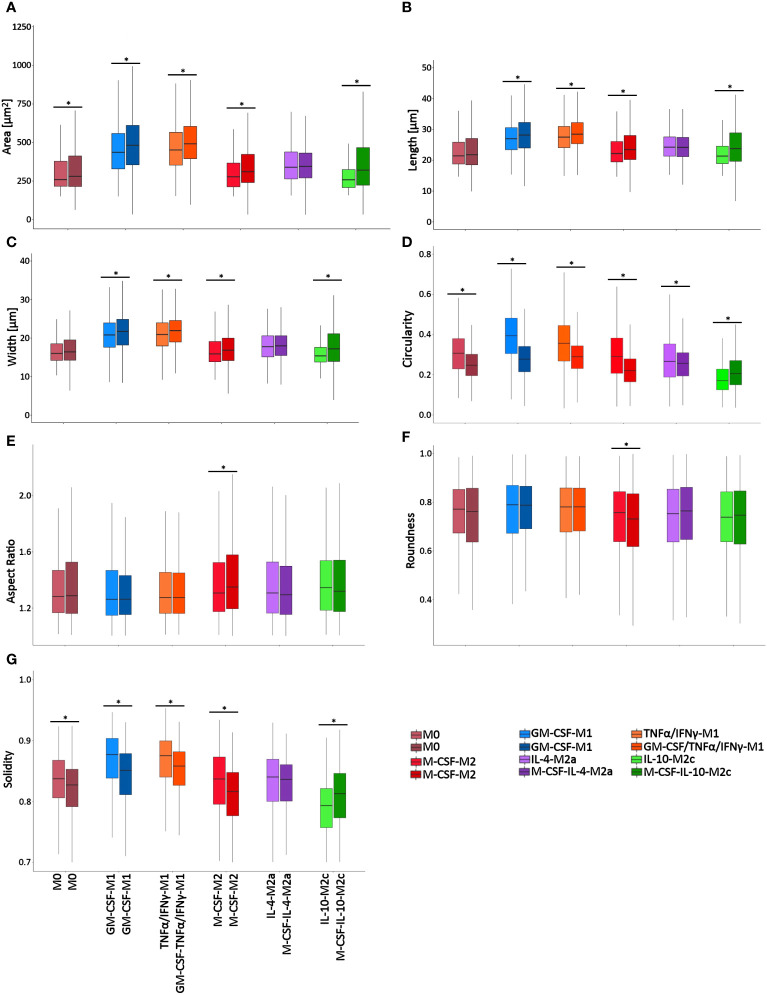
Single-cell macrophage morphology differs significantly between stimulation protocols/time points. Cell morphometric measurements of **(A)** area, **(B)** length, **(C)** width, **(D)** circularity, **(E)** aspect ratio, **(F)** roundness, and **(G)** solidity. The box plots present the shape descriptors of individually analyzed macrophages as a function of (i) the macrophage class and (ii) the stimulation protocol that was used. For each macrophage class, the left box plots are from Dataset 1 data, whereas the right box plots are from Dataset 2 data. Overall, the results confirmed that changes in the stimulation protocol significantly changed a range of single macrophage morphological features. *p<0.05. Boxplots: the boxes define the 25th and 75th percentiles, the central line indicates the median, and error bars define the 10th and 90th percentiles. *p<0.05. Dataset 1 consisted of 9304 cells in total, with n = 353 (M0), 3078 (GM-CSF-M1 control), 1891 (GM-CSF/TNFα/IFNγ-M1), 1321 (M-CSF-M2 Control), 1077 (M-CSF/IL-4-M2a), and 1584 (M-CSF/IL-10-M2c) individual cells analyzed of n=3 experiments per group, using 3 different donors. Dataset 2 consisted of 6072 cells in total with n = 279 (M0), 1768 (GM-CSF-M1 control), 1771 (GM-CSF/TNFα/IFNγ-M1), 903 (M-CSF-M2 Control), 922 (M-CSF/IL-4-M2a), and 399 (M-CSF/IL-10-M2c) individual cells analyzed of n = 3 experiments per group, using 3 different donors.

To validate the RF classification approach, we used a second, independent dataset (Dataset 2), which we generated using different stimulation conditions that were assessed on different days as opposed to the protocol of the first dataset (**Figure 1**). Macrophages were polarized in this second dataset in the absence of GM-CSF or M-CSF. **Figure 8** shows the resulting cell morphology of the different macrophage subtypes compared to the above-reported first dataset. Specifically, this change in stimulation protocol led to significant differences in the morphological features, including area, length, width, circularity, and solidity, whereas the cell aspect ratio and roundness remained constant, except for M-CSF-M2 cells. Notably, the morphology of M2a macrophages was mostly unchanged, except for their circularity. These data confirm that slight changes in the maturation or polarization conditions significantly changed macrophage morphological features, which indicated, in turn, that macrophage morphology is highly sensitive to both phenotype and stimulation protocol.

Next, this second original dataset was used to validate the chosen AI approach by generating synthetic data from this second original dataset. Subsequent RF modeling led to an accuracy of 95% for classifying all 6 macrophage phenotypes (**Figure 7C**, **Table 1**), which clearly demonstrated that the here chosen approach to classify macrophage phenotype, namely, using a large synthetic data set generated from experimentally measured cell morphological features as predictors, was able to reliably deliver high accuracy.

### 3.7 Assessment of the immunogenic potential of macrophages by predicting their intracellular IL-10 expression from morphology alone or from combined morphology and CD80 protein intensity

**Figure 9:**
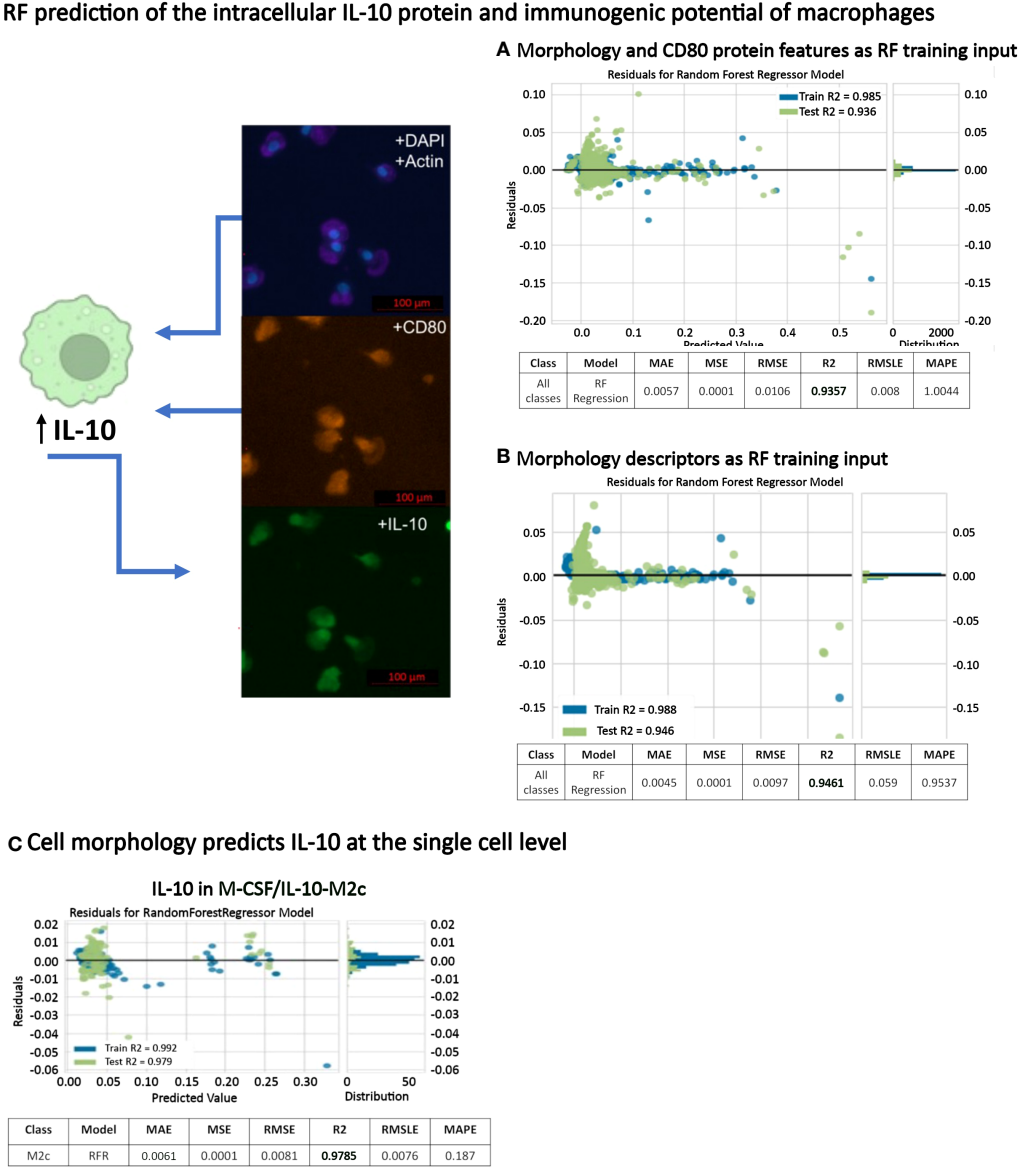
RF prediction of intracellular IL-10 protein expression of macrophages. **(A)** RF regression model based on morphology and CD80 protein intensity for predicting IL-10 protein intensity in all macrophage classes. **(B)** A second RF regression model using only morphology features as training input showed that cell morphology alone (without CD80 protein intensity) was able to predict IL-10 protein intensity in all macrophage classes with a high accuracy **(C)** RF regression model showing that the intracellular IL-10 content in M-CSF/IL-10-M2c macrophages can be predicted with a 98% accuracy using quantitative single-cell morphology features and AI.

IL-10 is a strong anti-inflammatory cytokine [26]. Here, intracellular IL-10 was expressed in all macrophage phenotypes to a greater or lesser extent, with high expression in M0, GM-CSF/TNFα/IFNγ-M1 and M-CSF/IL-10-M2c macrophages (**Figures 4F**, **6**). Because we achieved high macrophage phenotype classification accuracies based on macrophage morphological features (above), we further investigated the predictability of the IL-10 protein intensity (intracellular content) and, thus, the immunogenic potential of individual macrophages as a function of their phenotype class in a RF regression model; importantly, this has not been demonstrated before. In a first RF regression model, using single-cell shape descriptors combined with CD80 intensity data, we predicted the IL-10 intensity of all 6 macrophage phenotypes with a high R^2^ value of 94% (**Figure 9A**, **Table 2**). Further RF regression analyses of the individual stimulated classes revealed R^2^ values of 95% (M-CSF/IL-10-M2c), 93% (M-CSF-M2), 92% (M-CSF/IL-4-M2a), 85% (M0), 63% (GM-CSF/TNFα/IFNγ-M1), and 62% (GM-CSF-M1) classes, respectively.

**Table 2:** Summary of RF regression model performance to predict the single-cell IL-10 content (intensity).

RF Regression	Predictors	R^2^	MAE	MSE	RMSE	RMSLE	MAPE
All macrophage phenotypes	**Morphology + CD80 intensity**	0.9357	0.0057	0.0001	0.0106	0.008	1.0044
GM-CSF-M1		0.6367	0.0052	0.0001	0.008	0.0077	0.3955
GM-CSF/TNFα/IFNγ-M1		0.6187	0.0053	0.0001	0.008	0.0077	0.3994
M-CSF/IL-4-M2a		0.9164	0.0066	0.0001	0.0119	0.0107	0.2663
M-CSF/IL-10-M2c		0.9464	0.0075	0.0002	0.0128	0.0113	0.1981
M0		0.8543	0.006275	0.0001	0.0108	0.009	0.46705
M-CSF-M2		0.9314	0.00705	0.0001	0.0123	0.011	0.2322
All macrophage phenotypes	**Morphology alone**	**0.9461**	0.004	0.0001	0.0097	0.005	0.9537
GM-CSF-M1		0.7882	0.004	0.0001	0.0083	0.006	0.188
GM-CSF/TNFα/IFNγ-M1		0.7762	0.004	0.0001	0.0062	0.005	0.5269
M-CSF/IL-4-M2a		0.9462	0.006	0.0001	0.0096	0.009	0.3272
M-CSF/IL-10-M2c		0.9785	0.006	0.0001	0.0081	0.007	0.187
M0		0.8611	0.004	0.0001	0.0079	0.005	0.7403
M-CSF-M2		0.8824	0.004	0.0001	0.0083	0.006	0.63703

Note that the R^2^ values indicate the accuracy of regression.

Predictors are indicated in bold. This data highlights that quantitative single-cell morphology alone can predict intracellular IL-10 content in human monocytes (M0 cells) and five different macrophage phenotypes with a high accuracy (95% accuracy as indicated in bold).

After obtaining these excellent prediction results for IL-10 protein intensity using single-cell morphology and CD80 features, we investigated whether morphology alone could predict the macrophage IL-10 content. Importantly, the RF regression model predicted the IL-10 protein content of individual macrophages as a function of their six phenotype classes with a R^2^ value of 95% (**Figure 9B**). This was interesting because (i) here no synthetic data for increasing data set size was needed, and (ii) the IL-10 content prediction based only on morphology descriptors performed better than when CD80 co-staining data was included. The SHAP analysis, which informs the model user on the relative contribution of each feature to the overall model performance (i.e., indicates feature importance), demonstrated that cell area, length, and aspect ratio had the biggest impact. A further regression analysis of only the stimulated macrophage classes revealed R^2^ values of 95% for all 6 macrophage phenotypes, 98% (M-CSF/IL-10-M2c, **Figure 9C**), 95% (M-CSF/IL-4-M2a), 88% (M-CSF-M2), 86% (M0), 78% for GM-CSF/TNFα/IFNγ-M1, and 79% GM-CSF-M1 respectively. Therefore, these data show for the first time that by using only macrophage morphological features as predictors, successful prediction of single-cell intracellular IL-10 protein content with high R^2^ values is possible.

## 4 Discussion

Our study assessed the automatic classification of six distinct macrophage phenotypes, using image-based single-cell macrophage morphological features in two different datasets and both original and synthetic data. The datasets contained different phenotype morphologies induced by different stimuli. The first dataset was measured at day 4 and contained continuous presence of GM-CSF or M-CSF in combination with specific M1, M2a, and M2c polarizing stimuli. Validation was performed with data obtained on a different day (day 7) and using different conditions (polarizing stimuli alone without continuous M-CSF or GM-CSF) to test the model’s performance where conditions and, thus, resulting cell shapes and phenotypes can vary, as we proved by quantifying the differences in cell morphology. In both cases, high accuracies of 93% and 95% were achieved with synthetic training data for classifying macrophage phenotype original data. This confirmed that macrophage morphology is a highly sensitive dynamic marker that we used here for accurately classifying phenotype among six different phenotypes. Notably, single-cell morphometric features were also usable for accurately predicting intracellular IL-10 expression (R^2 =^ 0.95) and this was achieved without synthetic training data, indicating that macrophage morphological features are IL-10 content-specific, enabling successful prediction. Overall, this approach could potentially be used to discriminate, classify, and predict many more macrophage-related characteristics or expression profiles of any IL-10 producing cell.

The use of image-based machine learning using morphology-based features to accurately classify M0, M1, and M2 macrophages is in agreement with a previous study that showed a 90% accuracy using RF models to classify M0, M1, and M2 macrophages (9). However, our study extends this work and showed, for the first time, that image-based machine learning using morphology-based features could not only (i) classify M0, M1, and M2 macrophages but, more importantly, can additionally be used to (ii) classify M2a and M2c subtypes among six different phenotypes and (iii) additionally predict intracellular IL-10 at the single-cell level. That study (9) used a range of descriptors, measuring some of the same descriptors used in our study but also others. Here, we focused on seven cell-related cell shape descriptors without the need to include additional nucleus shape-related descriptors. In the present study, higher accuracies were achieved by increasing the dataset size through using synthetic data that was generated from the originally quantified data. This in turn suggested that the original dataset used to create the synthetic dataset was phenotype class-specific and, in short, “good enough”, otherwise the accuracy would not have increased despite increasing dataset size.

The panel of morphological descriptors that we used here was successfully used by our group to phenotype differentiated human mesenchymal stromal cells (MSCs) (30–33) and healthy vs. inflamed and degenerating diseased human chondrocytes (13). Moreover, using this panel as a phenotypic marker, combined with multivariate data analysis, we showed that the cell morphology and phenotype, i.e., the “biological fingerprint” of those inflamed and degenerated diseased human cells could be reverted to a healthier cell shape via therapeutic modulation and their healthier cell shape correlated with positive changes in major fibrosis- and inflammatory-regulating genes (29). Thus, our method provides a simple and cost-effective means of capturing cellular responses by quantitating cell morphology. In the present study, we used our recently introduced “SuperTiles” algorithm (39) to calculate synthetic data, including class-specific (aggregated) averages and (data enhancing) standard deviations with preserved inter-parameter correlations from randomly sampled and original datasets. The present study demonstrated that using this algorithm and the resulting synthetic training data increased the classification accuracy by 63% from 30% to 93%, which makes using synthetic training data for classifying original (measured) data a highly promising approach. Yet, even more encouraging was the successful use of a RF regression model to predict the intracellular levels of the anti-inflammatory cytokine IL-10 with original data only. Thus, we clearly demonstrated that a panel of cell shape descriptors was successfully used to reliably predict IL-10 content at a single-cell level (R2: 94%). In fact, the regression models trained on combined cell shape and CD80 expression were able to consistently predict IL-10 intensity with R^2^ values > 90%, but the inclusion of CD80 intensity data decreased the model performance, which was surprising. This could be due to marker variability, which is highlighted by the SHAP values for the M1 prediction model, whereby CD80 intensity contributed to both the model’s over-prediction and under-prediction. Importantly, the regression model produced and tested showed a strong potential to determine a macrophage’s inflammatory characteristics at the single-cell level based on cell shape alone. This might suggest a link between a cell’s morphology and some of its immunological functions.

Our study used standard conditions commonly used to generate distinct phenotypic macrophage states as they mimic different *in vivo* situations. In our first dataset, CD14+ monocyte-derived macrophages generated from peripheral blood monocytes were initially primed with GM-CSF (M1) or M-CSF (M2) followed by GM-CSF/TNF-α/IFN-γ (M1 macrophages), M-CSF/IL-4 (M2a macrophages) or M-CSF/IL-10 (M2c macrophages). These conditions were chosen for the following reasons. GM-CSF is produced under inflammatory conditions by a variety of leukocytes and other cells due to infection or injury and induces M1-like cells (34). GM-CSF alone can also induce differentiation of into dendritic cells (54, 55), which has not been examined here. Classically activated pro-inflammatory M1 macrophages have been known for some time to be induced by IFN-γ alone or in combination with TNF-α and GM-CSF (3, 5, 37). M-CSF is a homeostatic cytokine that is constitutively produced under homeostatic conditions and has been reported to induce M2-like properties (35, 36). But it is important to note that treatment with M-CSF alone may induce cells that stay at the monocyte stage if not additionally challenged with e.g., IL-4 or IL-10. In fact, an independent recent study using scRNAseq revealed that murine bone marrow monocytes cultured with M-CSF alone for five days remained at the monocyte stage with no or low expression of macrophage markers such as CD71 and F4/80 (56). Whereas our CIM results (**Figure 6**) showed that M-CSF-M2 cells are related and shared morphological features with M-CSF/IL-10 cells (M2c macrophages), suggesting M2-like properties, morphological assessment showed that the M-CSF-M2 cells were overall smaller, shorter, and rounder. Combining morphological assessment with immunological and/or biochemical validation could help clarify whether human peripheral blood-derived monocytes treated solely with M-CSF are more monocyte-like or partially or fully differentiated macrophages. Conversely, the alternative M2a macrophages, which have anti-inflammatory, wound healing, and pro-fibrotic properties, are induced by exposure to IL-4, whereas M2c macrophages, which have anti-inflammatory and tissue remodeling properties, are induced by exposure to IL-10 (M-CSF/IL-10-M2c) (3, 5, 37). The CIM plot revealed the complexity of data from these different phenotypes. Due to the size of the single-cell generated data, it was extremely difficult to identify specific patterns or clusters relative to the macrophage classes using single-cell data alone, which is why CIM with feature averages for each of the macrophage classes were additionally generated. This data revealed some interesting points, which have never been shown. For example, it clearly showed, based on hierarchical clustering, that both M1 macrophages, namely GM-CSF/TNFα/IFNγ-M1 and GM-CSF-M1 macrophages are related in both cell morphology and marker expression. This was supported by basic statistical analyses, which demonstrated that, for example, the M1 (both GM-CSF/TNFα/IFNγ-M1 and GM-CSF-M1) macrophages were similar in shape, except for their cell width and aspect ratio. The CIM results also showed that M-CSF-M2 and M-CSF/IL-10 (M2c macrophages) are related and share morphological features. Thus, the CIM with feature averages and, specifically, the associated hierarchical clustering revealed why AI was able to successfully and reliably classify six phenotypes with high accuracy, namely, because related macrophage classes, e.g. both M1 macrophages (GM-CSF/TNFα/IFNγ-M1, GM-CSF-M1), are related in their cell morphology.

Importantly, functional cytokine release of IL-6, TNF-α, and IL-10 into the culture supernatant (ELISA data) in combination with marker staining confirmed that the desired phenotypes were obtained and were similar to data reported in other studies (47–53, 57). However, when comparing secreted IL-10 vs. intracellular IL-10 expression, differences were noted. Results from our study showed that both intracellular IL-10 and secreted IL-10 were induced in M-CSF/IL-10-M2c macrophages in parallel with decreased TNFα production and increased CD163 expression. This is in agreement with other studies that have measured CD163 by other methods (i.e., flow cytometry) and protein secretion by ELISA (47–49, 57) in M2c macrophages. However, when intracellular IL-10 staining data (protein content) was assessed, significant levels of expression were also found in M1 polarized macrophages (GM-CSF/TNFα/IFNγ-M1) along with high CD80 expression as expected (47, 50–52) but, unexpectedly, these cells had secreted very little IL-10. While this initially appears contradictory to the expected results, M1 macrophages are known to stimulate IL-10 production in the presence of TNF-α (58, 59). Similar effects are seen in monocytes exposed to the bacterial endotoxin LPS (28, 58, 60, 61). This effect is specific to TNF-α and LPS and not induced by GM-CSF or other cytokines such as IFN, IL-1α, IL-1β, or IL-6 (58). This is in line with our results showing that the GM-CSF/TNFα/IFNγ-M1 macrophages but not GM-CSF-M1 macrophages expressed high intracellular IL-10. The results also showed that M0 cells (monocytes) also expressed relatively high intracellular IL-10, which is in agreement with flow cytometry studies measuring intracellular IL-10 or the gene expression of IL-10 in these cells (60, 61). Whereas the M-CSF/IL-10-M2c cells secreted high levels of IL-10, the M0 and GM-CSF/TNFα/IFNγ-M1 macrophages secreted extremely low levels of IL-10. This suggests that either we may have missed detection in M0 and GM-CSF/TNFα/IFNγ-M1 macrophages due to the timing when we measured IL-10 secretion, which was potentially too late since it was measured 2 days after polarization (half-life of IL-10: less than 1h (58)) or that the IL-10 protein reservoir was available but not yet secreted in these cells. Supporting the latter, it is important to note that the M1 macrophages used in our study were treated with IFN-γ, which was previously shown to suppress IL-10-induced secretion of IL-10 in RAW264.7 cells and bone marrow-derived macrophages (62), similar to what we observed in CD14+ monocyte-derived macrophages generated from peripheral blood monocytes.

Much of our understanding of how microenvironmental cues drive IL-10 production is based on ELISA or flow cytometry studies (24–28, 57) and very little has been reported on transcription to translation in relation to cell morphology. Whereas flow cytometry has been used to characterize intracellular IL-10 (24–28, 57), this is the first study to show that quantification of intracellular IL-10 can also be used to characterize polarized macrophages at the single-cell level and that morphological features can be used in turn to predict intracellular IL-10 protein content on the single-cell level. This new tactic may give rise to a novel way of assessing IL-10. Overall, this study adds to our understanding of morphology-related intracellular IL-10 expression in monocytes and macrophages and can help improve our understanding of cytokine biology at the single-cell level. Besides monocytes and macrophages, IL-10 is produced by almost all activated immune cells, including multiple T cell subsets, B cells, granulocytes (e.g., neutrophils, basophils, eosinophils), mast cells, dendritic cells as well as infiltrating and tissue-resident macrophages during disease or infection (23, 63, 64). Future studies will determine if this can be applied to these cells and possibly in more complex situations, such as in tissues and/or human disease.

In conclusion, our findings demonstrate a new image-based single macrophage classification method for macrophage phenotyping and characterizing intracellular IL-10, using solely cell shape as model input. Based on this simplicity, when paired with large enough datasets, this approach could become relevant for cell profiling in the context of *in vitro* studies or diseases known to involve macrophages and, in the case of IL-10, cell profiling of other cell types under inflammatory conditions or disease.

## Data availability statement

The raw data supporting the conclusions of this article will be made available by the authors, without undue reservation.

## Ethics statement

The requirement of ethical approval was waived by University of Freiburg Ethics Commission for the studies involving humans because peripheral blood mononuclear cells (PBMCs) were collected from three healthy human blood donors, who were all co-authors of this study. The need for approval of this study by the local ethics committee was discussed in advance with the committee. Due to the fact that only self-experiments by the authors were performed, under the relevant national and local regulations there was no obligation for approval. Therefore, after consultation with the local ethics committee, no vote was obtained. The studies were conducted in accordance with the local legislation and institutional requirements. The ethics committee/institutional review board also waived the requirement of written informed consent for participation from the participants or the participants’ legal guardians/next of kin because peripheral blood mononuclear cells (PBMCs) were collected from three healthy human blood donors, who were all co-authors of this study. Due to the fact that blood was only obtained from the authors, according to our local ethics committee (University of Freiburg Ethics Committe), under the relevant national and local regulations, informed consent is waived.

## Author contributions

MS: Formal Analysis, Investigation, Methodology, Software, Supervision, Validation, Visualization, Writing – original draft, Writing – review & editing. LP: Data curation, Formal Analysis, Investigation, Validation, Writing – original draft. NL: Methodology, Software, Writing – original draft. MV: Formal Analysis, Investigation, Methodology, Supervision, Writing – original draft. BR: Conceptualization, Data curation, Formal Analysis, Funding acquisition, Project administration, Resources, Software, Supervision, Visualization, Writing – review & editing. MH: Conceptualization, Data curation, Formal Analysis, Methodology, Project administration, Supervision, Visualization, Writing – original draft, Writing – review & editing.
